# Praying for a Miracle Part II: Idiosyncrasies of Spirituality and Its Relations With Religious Expressions in Health

**DOI:** 10.3389/fpsyg.2022.893780

**Published:** 2022-06-27

**Authors:** Marta Helena de Freitas, Miriam Martins Leal, Emmanuel Ifeka Nwora

**Affiliations:** ^1^“Religion, Mental Health and Culture” Laboratory, Catholic University of Brasília, Brasília, Brazil; ^2^Brasilia Maternal-Infant Hospital, Department of Neonatology and Catholic University of Brasilia, Brasília, Brazil; ^3^Saint Bonaventure Institute, Brasília, Brazil - Affiliated to the Pontifical Saint Bonaventure, Rome, Italy

**Keywords:** miracle belief, spirituality, religiosity, religion, psychology of religion, religious coping

## Abstract

As a continuation of the previous paper, *Praying for a Miracle – Negative or Positive Impacts on Health Care*, published in this research topic, this second paper aims at delving deeper into the same theme, but now from a simultaneously practical and conceptual approach. With that in mind, we revisit three theoretical models based on evidence, through which we can understand the role of a miracle in hospital settings and assess its impact in health contexts. For each of the models described, we seek to illustrate the possible outcomes of belief in miracles as a modality of religious coping in situations of stress and suffering experienced by patients and caregivers in the face of gloomy diagnoses on coming across the limits of medicine to revert certain illnesses (e.g., child cancer) or biological conditions (e.g., fetal malformation). We posit that the judgment about how such a mechanism is healthy or not for each of the people involved (patient, caregiver, and/or health professional) depends on the modulation between the conception of the miracle adopted by the patient and/or caregiver and the concrete outcomes of the way of responding to the situations that accompany the gravity of the illness or condition. To better understand this process of psychological modulation that accompanies belief in miracles, we revisit the concepts of spirituality, religiosity, and religion, pointing out the connections and distinctions between them from a phenomenological perspective. We then present a conceptual model that takes these connections and distinctions into consideration to foster an understanding of miracles, their relations with the diversity of experiences of people who meet in hospital settings (patients, caregivers, and health professionals), and their respective impacts on healthcare.

## Introduction

The theme of miracle, scarcely, or rarely discussed in the scientific circle of medicine and psychology, is significantly constant in daily hospital experience (Ellington et al., 2017; Saad and Medeiros, 2018; Shinall et al., 2018; Bibler et al., 2020; Gradick et al., 2020), especially in situations that involve diagnoses, for which there are no hopes for a cure by the medical team. Even though the belief or hope in miracle is not a privilege of only the patient and caregivers, and may also be nurtured by health professionals—especially in countries with a high religious population like Brazil, for example, studies have pointed out strong resistance, enormous difficulties or, in the least, some discomfort in handling the question by members of the medical team (Green, 2015; Rossel et al., 2016; Ellington et al., 2017; Freitas et al., 2017). Even though many professionals frequently attribute such reactions to the supposedly (de)negating component of miracles, which would hinder the confrontation of the reality associated with the gloomy diagnoses (Carlsson et al., 2017; Borges and Petean, 2018) and would induce wishful thinking (Vasconcelos and Petean, 2009), the question is much more complex.

As demonstrated in another paper of this same research topic - *Praying for a Miracle, Part I* (Leal et al., 2022), the simplistic perspective of the notion of a miracle may pave the way to understanding it as an extraordinary event not applicable to the laws of nature, and, consequently, to the interpretation that the hope (by patients and caregivers) of its occurrence would necessarily imply the refusal of recognizing and assuming the gravity of the organic conditions resulting from illnesses, traumas, accidents or whatever concrete limitations scientifically diagnosed by medicine. Nevertheless, such a reductionist conception, much common among medical team members; sometimes reinforced by psychological theories that created the stereotype that religiosity and religion are coping strategies focused only on emotion (Pargament et al., 2005), on phantasmagoric illusion (Ghsymala-Moszcynska and Beit-Halahmi, 1996), or alienation from reality (Chagas, 2017), maybe a disguise that absconds graver problems. As such, from workplace mental health and the psychodynamic point of view, the conceptual reductionism of the notion of miracle and the elements that sustain it can be interpreted as symptoms of a kind of defensive ideology, in the sense defined by Dejours (2018), shielding medical team members from direct contact with their existential dilemmas, as well as their impact on medical practice, and/or with situations that might be out of their objective control. From the practical point of view, it could be an exposure to a lack of scientific (or even theological) knowledge by a great majority of health professionals about the role of spirituality and religiosity on physical and mental health (Koenig, 2012; Filho et al., 2021). This often aggravates the insufficiency of relational and sociocultural competencies (Whytley, 2012; Swihart et al., 2021) in handling overwhelming questions that are beyond the objective limits of medicine, such as those associated with the finitude of life and its mysteries, for example.

In light of the above, there arises the necessity of a deeper discussion of this question in scientific settings and in an interdisciplinary perspective, where the psychology of religion, in dialogue with other fields like theology, medicine, and philosophy, can contribute to a better understanding of the psychological aspects involved in the belief in miracles and its impacts on physical and mental health, either for patients, caregivers or for health professionals themselves. This paper, therefore, in conjunction with the aforementioned study by Leal et al. (2022), aims at contributing to this end. The first study focused on the problematization of the dichotomy around the positive or negative role of the belief or hope in miracles, and presented a critique grounded on a phenomenological perspective, pointing out the risks of a conception that hastily judges them as harmful to patients' (or caregivers') physical or mental health – and as such, tags them as negative spiritual/religious coping (NSRC) strategy. This second study, *Praying for a Miracle – Part II*, in its turn, aims at deepening the subject from an approach that is simultaneously practical and conceptual. Therefore, three models based on evidence already presented in the previous paper (Leal et al., 2022) will be explored here from a phenomenological perspective and, posteriorly, placed in dialogue with the concepts of spirituality, religiosity, and religion. It is assumed that such a perspective provides an axiological and epistemic grounding capable of offering a theoretic-conceptual underpinning that would permit health professionals, including the psychologist, to situate the experience of believing in a miracle in a complex network of meanings, symbolisms, and existential meanings, and understand its relations with the human dimensions of spirituality, religiosity and religion, and the respective connections between them. The starting point is the principle that such an understanding, besides fostering a constant exercise of auto reflexivity by professionals, will offer subsidies that enhance a more sensitive and adequate clinical management of the various ways, through which belief in miracles is effectively manifested in hospital settings.

To achieve these objectives, this paper is laid out in the following manner: after this short introduction, we present three models based on the evidence currently proposed for understanding the role of miracle, derived from the effort to understand its idiosyncrasies in specific contexts, including those involving children in grievous states and their family members. We, then, discuss from the historical and epistemological point of view, the connections and distinctions between spirituality, religiosity, and religion, presenting a conceptual model based on phenomenology. From this model, we seek to understand the modalities and possible outcomes of belief in miracles (as an expression of spirituality, religiosity, and/or religion) in hospital settings, and discuss the outcomes for clinical handling in concrete situations of healthcare.

## Proposed Models for Understanding the Role of Miracles in Health Settings

One of the models already proposed for understanding belief in miracles in health contexts is directly derived from the conception of spiritual/religious coping (SRC) elaborated by Pargament and his collaborators (Pargament and Hahn, 1986; Pargament, 1990; Pargament et al., 1990). As pointed out in the previous paper by Leal et al. (2022), such a model, contributes to a pragmatic approach to the subject, promoting studies of nomothetic nature in the field of psychology of religion and investigating SRC on large scales, led to a dichotomous classification of miracle into positive and negative. This is well illustrated, for example, in the elaboration of the North American SRC scale (called RCOPE), created by Pargament et al. (2000), and later, translated and adapted to Brazil by Panzini and Bandeira (2005). In this instrument, SCR was divided into eight factors characterized as positive (PSRC) and four factors characterized as negative (NSRC). Even though in this instrument, the act of praying for a miracle was classified into the group of negative factors, in the analysis of Leal et al. (2022), this was due to a reductionist conception of the act in question, classifying it as a coping strategy focused on emotion and characterizing a passive religious delegation or negative prayer for tending to modify divine will. It is worth mentioning that even from a cognitivist perspective, Pargament himself and some of his collaborators, in more recent works (e.g., Pargament and Exline, 2022), have used a less dichotomous terminology to refer to SRC, for example using the expression “religious/spiritual struggles” instead of the notion of NSRC.

From a phenomenological point of view, we can understand that the cognitive model developed by Pargament and collaborators (Pargament and Hahn, 1986; Pargament, 1990, 1997; Pargament et al., 1990), if understood from a more qualitative perspective and less committed to a classificatory and dichotomous concern, allows for an evaluation of the psychological function of the belief in miracles, which is also understood from a broader phenomenological conception and not merely reductionist, about each one of the factors mentioned in the scale. For a better understanding of the exposition made above, we make in a concise form an application of this model in the evaluation of the act of praying for a miracle, considering each of the factors, positive and negative, mentioned in the scale (called RCOPE).

The seven factors originally classified as positive in the scale elaborated by Pargament et al. (2000) were transformed into eight in the version translated and validated to Portuguese by Panzini (2004). They are as follows: (1) Transformation of self and/or of life, by which it could be evaluated how belief in miracles can bring about or not, a personal transformation, either in the form of an internal and/or external modification of life; (2) Actions in search of spiritual help to aid in assessing the role of hope in miracle in the move of seeking in the other (individual, institutional, family, or social) a kind of spiritual help, e.g., spiritual treatment, orientation from spiritual entities, reposition of vital energy, and practice of activities in search of spirituality or more connection with it; (3) Offer of help to others, by which to identify how belief in miracles can foster behaviors of helping the other (individual, institutional, family, or social), either through prayers, support and/or spiritual orientation, donations, voluntary work, and/or affective-cognitive internal modifications for the benefit of others; (4) A positive stance before God, a factor that permits the assessment of how the behavior of praying for a miracle reveals or not a personal attitude before God about the situation, manifests either through religion, a search for support in God, or more connection with Him and/or of positive evaluations through Him and manifest in such attitudes, as collaborating, praying, approaching, counting on and/or depending on God, or individual actions independent of God's help; (5) A personal search for spiritual growth, by which to assess show much hope in miracles reveals a personal search for God and/or of spirituality (in contrast to institutional search), or a search for self through God and/or of spirituality, capable of being manifest, for example, through positive reassessments, non-institutional practices, search for deep connection with self or with forces that transcend the individual; (6) Actions in search of the institutional other, that can reflect how much the belief in miracles enhances the move of approaching religious institutions, places, members or religious representatives, or other manifestations that might result in support and institutional belonging; (7) A personal search for spiritual knowledge, through which to judge how much belief in miracles enhances more religious-spiritual knowledge, with the aim of, for example, internal self-strengthening in confronting the world and/or divine plans, increasing religious practice, or developing new attitudes in life; seeking help to cope and/or understand the situation, resulting in intellectual growth, among others; and (8) Estrangement through God, religion, and/or spirituality, a factor through which to distinguish how belief in miracles promotes a change of personal perspective about the situation, in which a person distances him or herself from the problem and respective stressful condition to approach God and/or religious/spiritual questions, without necessarily characterizing mere avoidance, but only a temporary distancing and capable of permitting him or she oxygenizes the mind, on focusing attention on another subject, specifically, on spiritual and religious aspects.

The four negative factors mentioned in the same scale (Pargament et al., 2000; Panzini, 2004) are (1) A negative reassessment of God, that permits an evaluation of how much belief in miracles in health contexts would result in a negative cognitive reassessment of the person's idea of God, raising disturbing interrogations about Him and His plans, for example, distressing doubts about His existence, power, love, protection, as well as about His responsibility regarding the gravity of his or her illness, which can be interpreted as divine punishment, thereby, harboring negative sentiments like anger, guilt, helplessness, and grievance; (2) Negative stance before God, by which the manifestation of the act of praying for a miracle can be assessed in the measure that it implies a request or simply hope that God takes control of the situation and takes up the responsibility of solving the problem without his or her participation, which could be expressed, for example, of the passive religious delegation or negative prayer, when prayer simply tends to modify the supposed divine will; and (3) Negative reassessment of meaning, according to which the attitude of praying for a miracle, in hospital settings, can be assessed in the measure that it results in interpreting negatively the meaning of the gravity of the illness as an act and/or consequence of Evil or as a punishment for his or her acts, style of life, errors or sins. In this last case, then, Evil would necessarily be associated with a personalized being figured as Demon, Devil, Satan, and Beelzebub, among other denominations; or to an abstract figure, like darkness, gloom, dark side, or Evil itself; or still, incarnated in figures that practice such evil, like evil spirits, forces of darkness, ill luck and/or other peoples' evil wishes to him or her. However, whatever the reason for falling into the grave illness without any perspective of medical treatment, it would be understood by the patient or caregiver as personal punishment or the result of something evil. (4) Dissatisfaction with the Institutional Other, by which every behavior or SRC attitude, including praying for a miracle, would be assessed in the measure that it reveals sentiments of dissatisfaction, displeasure, or grievance with any institutional representative, be it a frequenter, member, or leader of the religious institution or symbolized by the set of religious or spiritual doctrines of the person.

It is, therefore, imperative that, for a concrete assessment *in loco* about the role of belief in miracles to be made according to the degree of its correspondence or not to each of the factors enumerated above, it will be necessary to assume a broader conception of miracle like that of Tillich (1967) or Saint Thomas Aquinas (1265–1273) for example, as was seen in *Praying for a Miracle – Part I* (Leal et al., 2022). In such a perspective, the miracle is taken as a kind of “signal-event,” and which occurs as a reflection of something divine, but also natural, both from the point of view of aligning with the laws of nature—although inaccessible or completely inexplicable by scientific knowledge produced by humanity, and from the psychological point of view—based on the dialogue/communication between the desire of overcoming the condition imposed by the grave illness and another dimension superior to this desire, and which is lived as a sacred or divine order. It depends, therefore, on the modulation between the conception of miracle adopted by the patient and/or caregiver and the concrete outcomes of his manner of reacting to situations that accompany the gravity of his or her illness to assess how belief or hope in that “signal-event” will be healthy or not for each person involved; patient, caregiver and/or health professional. After all, as shall be seen later, the conception of miracle is experienced in conformity with the way that the person internally elaborates his or her dynamics of the search for existential meaning (spirituality) and how this answer is searched for in the transcendent (religiosity), which can be anchored or not in a specific religion.

Considering the modulation referred to above and its concrete reflections in health settings, we can understand the implications of belief in miracles from the standpoint of a second model, as proposed by Shinall et al. (2018), and elaborated from the concrete results observed, for example, in palliative care settings. As was seen earlier, in *Praying for a Miracle – Part I* (Leal et al., 2022), the above-mentioned authors classify the various modalities of belief in miracles into at least four patterns: (1) “Innocuous”, which occurs when motivated by a belief in miracles, the patient or caregiver expects a plausible positive result, but improbable for a cure, without, however, sparking off conflicts with the health professionals monitoring the case; (2) “Shaken hope”, when faith in miracle becomes hampered as a result of unfavorable clinical evolution, which does not often trigger conflict with members of the medical team, but spurs significant existential pain, affecting the quality of life of the patient; (3) “Integrated”, when belief in miracles is based on religion and can be in discord with health professionals and ignite conflict in the doctor-patient relationship; and (4) “Strategic”, when the patient's or caregiver's belief in miracles is characterized as one of the ways of affirming his or her power over the situation and impedes deeper discussions about medical intervention decisions, and thus being interpreted by health professionals as a negation of reality. It should be observed that, in this second model, the criterion of assessment of belief in miracles is not restricted only to the analysis of the characteristics of the patient or caregiver, but also takes into consideration its impact on the health professionals and their respective reactions. It is important to consider this in the measure that the reactions of the professionals also depend both on the way that they feel more or less mobilized about the specific way that their patients' belief in miracles is manifest, and also on the way they internally elaborate their own experiences related to spirituality, religiosity, and religion.

For assessing the impact of belief in miracles in health settings, a third model of a pragmatic nature was also developed from clinical experience and, especially, more applicable to caregivers of gravely ill children was developed by Bibler et al. (2020). According to this model, the ways by which beliefs in miracles can be assessed, from the standpoint of their concrete outcomes in healthcare settings, could be classified into three modalities: (1) “Integrated,” when caregivers assess the clinical state of the child from a religious standpoint, making them not only carry religious objects in infirmary settings, but also, frequently establish confrontation with science; (2) “Procurators,” when such caregivers depend on the religious community, but the miracle hope may assume meanings other than the belief of obtaining cure, for example, focusing on the wellbeing of the child; and (3) “Adaptable,” when they present characteristics of having faith, without necessarily adhering to specific religions. In this last case, the caregivers generally, avoid talking about the miracles, but, on the other hand, are also suspicious of the strictly technical care given to patients, and may have recourse to other options in search of a cure for their loved ones.

One of the aspects common to the three models presented hitherto is the fact that all of them, in one way or the other, refer to three concepts that, generally, have been interchanged not only in lay discourse, but also in the technical language of the psychology of religion, be they concepts of “spirituality,” “religiosity,” and “religion.” In fact, in the field conventionally called “psychology of religion,” frequently renamed “psychology of spirituality” (Aletti, 2012; Paloutzian and Park, 2012; Pargament et al., 2013a; Freitas, 2017), there is a great conceptual variety, especially about the three terms mentioned above. They have received a more integrative, interdisciplinary, and multidimensional approach in recent years (WHOQOL Group, 1995; Pargament et al., 2013b; Selvan, 2013), considering their applicability in health settings and allied to the perspective that health cannot be reduced to the biological dimension or the mere absence of disease, but encompassing psycho-socio-spiritual aspects that are fundamental to wellbeing as advocated by the WHOQOL Group (1995).

Our starting point in this paper is the principle that, despite the inherent complexity of the terms, and the lack of consensus between diverse authors, the distinction between spirituality, religiosity, and religion, as well as their respective integrative approaches, are not only useful but also necessary and fundamental for a contextualized and dynamic approach of the different meanings of belief in miracles in the spiritual/religious coping with illness, stress and psychic suffering. In this light, the next sub-item, of a more historical, conceptual, and philosophical nature has the aim of presenting an epistemological grounding to understand the possible connections and distinctions between the three terms. By doing so, we hope to foster a deeper understanding of the complex nature of belief in miracles and their outcomes in people's lifeworld (*Lebenswelt*).

## Connections and Distinctions Between Spirituality, Religiosity, and Religion and Their Relations to Health

A brief incursion into the works of great pioneers in the psychology of religion shows that, originally, the terms spirituality, religiosity, and religion were employed in much a complementary or practically undifferentiated way (Zinnbauer and Park, 2005; Freitas, 2017). However, with the advent of modernity, along with the processes of secularization, secular state, globalization, individualization of manners of spiritual-religious expression, and the demand for the applicability of methodological reduction of the transcendent to the psychology of religion and/or spirituality (Flournoy, 1902), so that it is recognized in the postmodern scientific scenario (Paloutzian and Park, 2021; Saroglou, 2021), there was an emergence of a conceptual landscape characterized by a veritable increase of differences between these terms, especially between spirituality and religion. Thus, it is common to find negative reports of religion and positive reports of spirituality coming from both researchers in this field and physical and mental health professionals (Freitas, 2020). In the same way, the expression “I am spiritual, but not religious” (Maraldi, 2014, 2022a,b), is frequently reported in interviews with psychologists and health professionals (Freitas et al., 2017; Freitas, 2020) has become more common, and paradigmatically represented in the work of the famous neuroscientist Harris (2014), titled *Waking Up: A Guide to Spirituality Without Religion*. The deadlocks created by this dichotomy and terminological polarization are numerous, and as a result, many authors have been critical and opposed to them (Zinnbauer and Park, 2005; Aletti, 2012), and trying to propose solutions to overcome them. However, these solutions, also, are not convergent (Zinnbauer and Park, 2005; Marques, 2010; Aletti, 2012), and oscillate between those that: (a) adopt a perspective of partial convergence between the religious and spiritual dimension; (b) understand religion as a more far-reaching field than spirituality; (c) understand spirituality as a more far-reaching field than religion; (d) differentiate both by the intensity and emotional involvement, with which they were lived; (e) maintain an absolute distinction between both and see them as opposed dimensions.

Relatively, the term “spirituality”—a concept of much fluid origin, derived from the Latin term *spiritus* and etymologically referring to the notion of “breath of life” (Hill et al., 2000; Carrette and King, 2004)—tends to be intimately associated today to the conception of purpose or meaning of life (Aletti, 2012; Piedmont, 2014; Cook, 2020; Freitas, 2020), and does not have any consensus among the authors. The contemporary scenario is paradoxical: concomitant to the plethora of studies and applications of the construct in clinical settings (Cunha and Scorsoline-Comin, 2019; Demir, 2019; Cook, 2020; Dein et al., 2020) are an endless barrage of stringent epistemological criticisms of the way that spirituality has been conceptualized and re-conceptualized in studies developed in physical and mental health settings. Examples of such criticisms are those that point to the excessive generality of the term, loaded with polysemic and vague meaning (Carrette and King, 2004; Paiva, 2004; Swinton and Pattison, 2010; Aletti, 2012) and also those that point to its tautological character when defined as almost a synonym of mental health, wellbeing or positive psychological or social traces in studies that aim at correlating spirituality to these same variables (Almeida and Koenig, 2010; Koenig, 2010; Curcio et al., 2013).

Another grave risk, that must not be ignored from the phenomenological-existential point of view, is that of the efforts to reduce the ambiguity of the term spirituality resulting also to its impoverishment and eroding of its original meaning (Freitas, 2017; Silva and Goto, 2020) such as genuinely experienced by people in their life world. In this sense, attention must be paid to contemporary contradictions: if, on one hand, the operationalization of the concepts enhances their application and validation in studies and actions that might be recognized in the field of health, on the other, this is accompanied by the risks of a grave alienation of that which is intended to be valued on bringing the subjects of religiosity and spirituality to this field.

The term religion, also derived from the Latin *religio*, even though it tends to evoke, immediately, complex representations that refer to the relationship of man with the transcendent, its original meaning is debatable from its etymological roots as rightly pointed out by the philosophers of religion Costa Freitas (1992) and Azevedo (2010). Thus, in the Roman version of Cicero, the term would date back to the notion of *relegere*, meaning something like “revolve in spirit, care for, take seriously, meditate”, or still “decency and retreat, scruple and delicateness of conscience, fulfillment of duty to things and people, worship of the gods (Costa Freitas, 1992, p. 676). But in the Christian version of Lactantius and Tertullian, *religion* would be derived from the verb *religare*, therefore, referring to the attitude of devotion and piety that unites men to God. The notion of bond is highlighted here, a kind of rebinding, between humanity and a power that transcends it (Hill et al., 2000; Azevedo, 2010). The innumerous historical attempts to define religion are, therefore, situated in this semantic horizon and some currents opt for a more functional conception while others are characterized by a more substantive conception. Both approaches converge in the reference to a transcendent dimension, worshiped in form of laws, norms, doctrines, and/or moral rules, among other things, or experienced as a true existential response to the great questions of the meaning of human life. A phenomenological look leads to understanding the phenomenon of religion as a subjective/intersubjective process with attitudinal, communal, and institutional ramifications. Some part of contemporary psychological literature tends to attribute to the first of a more subjective order, the concept of religiosity (Valle, 1998) or intrinsic religiosity and/or not organizational (Koenig, 2011); while, for the second, communal and institutional, it tends to reserve the terms religion (Valle, 1998) or organizational religiosity (Koenig, 2011).

On distinguishing the subjective and institutional level of the religious phenomenon, there are still, in literature, other dichotomous tendencies in the conceptual framework, including those that refer to their various forms of expression in the life world of people, forms that have been frequently classified between two groups, from the standpoint of substantive or pragmatic criteria. Examples of these are the classification of Allport (1950) into intrinsic and extrinsic religiosity, and the contemporary classification of Pargament et al. (2000) into positive and negative forms of religious coping, both originally from qualitative assessments, but later served the purposes of nomothetic studies, as the scale of intrinsic and extrinsic religiosity (Allport and Ross, 1967) and the scale of religious coping (Pargament et al., 2000). If the driving impact of such instruments is undeniable in the sense of allowing correlations between the multiple expressions of religiosity and many other variables associated with health, contributing to more credibility of Psychology of Religion in scientific circles, their excessive and indiscriminate use, frequently in ways completely dissociated from the epistemological foundations that gave its origin (Forti et al., 2020) and from the sociocultural characteristics where it is transplanted, also created problems, between them: the risk of an erosion of the notion of spirituality and its relations to religiosity (Silva and Goto, 2020), and the tendency of an artificial dichotomization between its various modalities of expression and respective dynamism. In *Praying for a Miracle – Part 1* (Leal et al., 2022), we see the vicissitudes of this process in the assessment of belief in miracles through the RCOPE scale (Pargament et al., 2000) and its respective translation for Brazil (Panzini and Bandeira, 2005), where the modality of spiritual/religious coping was previously associated to negative religious coping.

There is still another important ethical and epistemological outcome for the study of the understanding of miracles from the psychological point of view in health settings: the necessity of differentiating a theological approach from a psychological approach, respecting the dialogue between both, and with due openness to the contributions of other sciences, for example, medicine, in an interdisciplinary perspective. This implies recognizing that the religious cannot be reduced to the psychological, in the same way, that the study of the ontological reality of the transcendent cannot be attributed to psychology, but rather, the human experience with the transcendent. In other words, psychology cannot affirm or deny the objective existence of God, Jehovah, or whatever term that may be used to designate any kind of sacred or transcendent alterity. However, it is its responsibility to focus on the understanding of the human experience with the alterity in question. To put it in Husserlian terms, psychology should focus on the “lifeworld” (*Lebenswelt*). This, taken as a focus by scientific knowledge, should not be reduced to the point of completely losing it from sight (Valle, 1998), but, at the same time, in the case of religious experience, the demand for methodological reduction of the transcendence applies, e.g., as proposed by Flournoy (1902). In the case of studies directed toward belief in miracles, for example, this ethical demand implies necessarily qualifying the experience with the transcendent, as something that is constituted in the conscience of who lives it, but also suspending the ontological reality of the transcendent alterity (God, in Western society), considering it outside of the possibilities of hermeneutic recognition. This same demand should be placed before the health professional, especially in countries officially governed by the principle of a secular state, like Brazil. However, in the name of this principle, human experience, including belief in miracles, should not be reduced to mere medical or psychological categories previously established. This would amount to exercising secularism instead of secularity (Ranquetat Jr., 2008).

In fact, from the ethical and phenomenological point of view, the psychology of religion and/or spirituality should analyze human experience in its richness and diversity, be it of a subjective, intersubjective, social, and/or cultural nature, assessing not only the experiences of those that describe themselves as religious but also of those who regard themselves as spiritual but not religious, a reality that is more and more common in contemporaneity. In other words, such people often admit that they are propelled by the search for answers that should significantly satisfy their thirst for existential meaning, but assume that this thirst for meaning is not adequately satisfied through faith in the transcendent dimension. For some of them, this thirst is satisfied through art; while for others, through contact with nature, profession, philosophy, and/or science, and this often assumes for these people a “sacred” character. To legitimate, their experience implies defining spirituality in such a way that “suspends” the origin of “breath” that impels them in the search for meaning. This can be applied to the experience of many health professionals, especially doctors and psychologists who practice in health and hospital contexts – though not all, as many believe! (Freitas, 2020). In these cases, it becomes important to consider a conception of spirituality that does not make a pronunciation about the previous and founding ontological reality of the same dynamics of the search for existential meaning. In other words, it demands a definition of spirituality that does not negate and neither affirm its divine origin, but at the same time legitimates the search for meaning in life which also propels the life world of these people, though not necessarily finding their answer in the belief in God or in some other dimension that is culturally equivalent. That is to say, for these people, the essence of spirituality is concretized in the move of the search for meaning and not properly like the answer found through belief in the transcendent. For many of them, the answer will be found in the contact with nature, art, philosophy, professional practice, or science. At least, they describe themselves in this manner.

**Figure 1:**
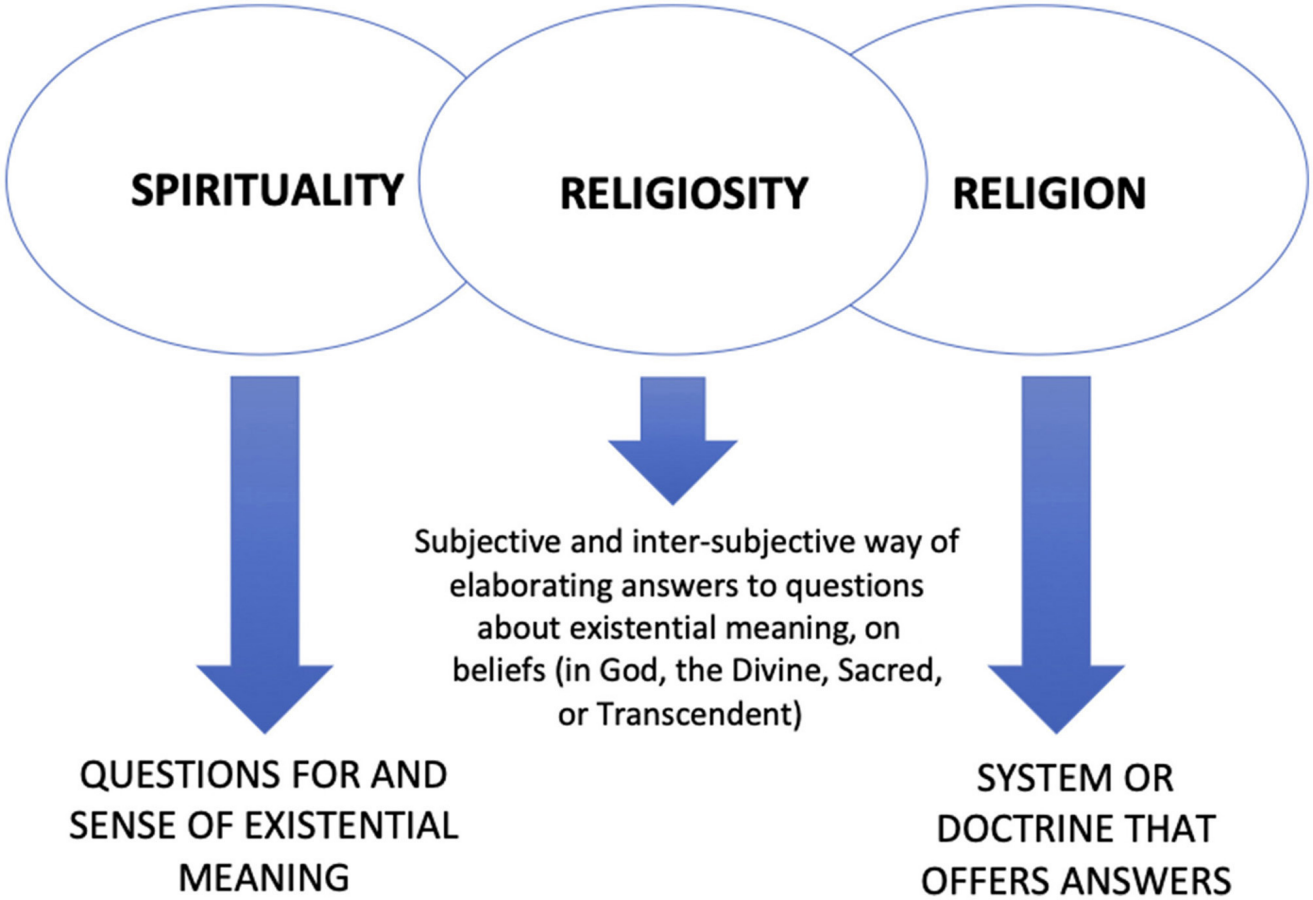
Concepts of spirituality, religiosity and religion based on phenomenology. Reproduced and adapted from Freitas and Vilela (2017, p. 97).

In light of the above, it is fundamental to find a conceptual model in the psychology of religion that considers the diversity of experiences (of patients, caregivers, and health professionals) that come across each other in hospital settings, in such a way as to enhance an understanding of the impact (positive or negative) of the belief in miracles in healthcare. The conceptualization of spirituality, religiosity, and religion in this model should, therefore, foster a simultaneously distinctive, integrative, and qualifying understanding of these three phenomena and their manifestations in human experience, be it that of patients or health professionals. A simplified alternative, though not reductionist or dichotomous – of defining the three terms and presenting their complex interrelations is illustrated in Figure 1, reproduced from Freitas (2020, p. 204). It should be observed that the model proposed is open enough to compose the experiences of those that consider themselves spiritual but not religious as well as that of those that nurture a personal religiosity, not necessarily adhering to a specific and institutionalized religion. Meaning, in the same way, spirituality can move in the direction of other answers of meaning not peculiar to religiosity, the religious experience can also occur in the subjective domain but does not necessarily seek to be anchored or aggregated to a system of organized answers in the mode of dogmas or doctrines and/or institutionally shared.

Grounded on a phenomenological perspective, the model takes spirituality in the Husserlian sense and refers to an existential quest for meaning, but directed “exclusively to human beings as persons, to their personal life and activity, as also correlatively to the concrete results of this activity.” And, as says Husserl (1965, p. 1): “Here the word ‘live' is not to be taken in a physiological sense but rather as signifying purposeful living, manifesting spiritual creativity, in the broadest sense, thereby creating a culture within historical continuity.” Thus, it situates spirituality in the original pole of the great questions about life, and existence, and is often formulated by the common person –but also by the religious, philosopher, or scientist—in this way: “where did we come from”?; “where are we and what are we doing here”?; “where are we heading to”?. As it is well known, even though such questions are a constant part of human existence, they become more overwhelming in situations of crises and great suffering, e.g., facing grave illnesses, gloomy diagnoses, and the finitude of life.

To effectively understand spirituality, it is necessary to understand also how it propels those who practice it. Thus, belief in a transcendent, sacred, creating, infinite, ultimate, or beyond the human has constituted a kind of response that accompanies humanity historically and geographically in all known cultures. This way of responding to the quest for meaning is given the name religiosity, which may or may not is shared collectively as happens in religion. The answers to the great questions about meaning can naturally be sought in various other ways, either through contact with nature or art or through philosophy or scientific activity as was previously pointed out. Nevertheless, a significant majority, especially the Brazilian population (Instituto Brasileiro de Geografia e Estatística., 2010), are crystallized in religious adherence. So, in the face of adverse, limiting, and incomprehensible conditions capable of threatening existential meaning, these people, mobilized by the search for the meaning of their experiences, seek answers for their interrogations in a dimension that is beyond them, nurtured by the belief in a being, force or superior energy, capable of responding to them and bring consolation, comfort, serenity, resilience, and/or hope.

When the ways of finding answers to existential interrogations through religiosity are collectively shared, in an institutionalized way, forming a social hierarchically organized entity, or characterizing a cultural identity of a people (as it is the case of indigenous people for example), the concept of religion is applied. The experiences of religiosity and religion, therefore, constitute religious people's experiences, through which connectivity to the response is attained, propelled by the quest for meaning, in other words, by spirituality. Besides, in addition to the answers offered by institutional dogma, systemized doctrines in specific modalities of belief and value, normative and behavioral guidelines, or leaderships that represent them, is the psychological role played by the feeling of belonging, reception, and support offered by the network of adherents of the doctrine in question, besides a series of other outgrowths that delineate the features of the quest for meaning, alleviating grief, anxiety, helplessness, and despair, that is frequently unleashed by unexpected, limiting and disastrous news, including naturally, gloomy diagnoses received in hospital settings about one's health or that of a loved one.

## Belief in Miracles as an Expression of Spirituality, Religiosity, and/or Religion

The questions of a conceptual nature about the experiences said to be spiritual or religious are important for an understanding of the belief in miracles and their impacts on the actors in hospital settings, not in the sense of arriving at a final decision about what would be a “correct” conceptualization of what spirituality, religiosity, religion, or miracle itself is. Instead, they are important for their pragmatic outcomes, either in the field of research, permitting a clearer communication about these questions, or in terms of its concrete applications in healthcare. It is, therefore, from this perspective that we aim to direct the reflections stemming from the conceptual model previously proposed and its respective applicability for understanding belief in miracles and its respective role in health settings.

The notion of spirituality as a propelling force in the quest for existential meaning makes it possible to think of health, from a broader perspective, where at stake is not only a biological body or medical rationality restricted to the technical operationalization of the human, whereby the physical, biological and material aspects become prominent over social and teleological aspects. Now, a more far-reaching understanding of health makes existential liberty necessary both for patients, caregivers, and professionals, to own up to themselves in their existential questions and respective quests for meaning, and at the same time permit them to be more sensitive to the different ways and paths by which the other also makes the search. It is within the scope of this same liberty and sensitivity that belief in miracles can be recognized as one of the possible destinations in the natural quest for meaning. In this existential move of propelling hope, we can understand its dynamic character, the motivation for searching for meanings and purposes, how the person connects with him/herself, with illness, with the other, with the cosmos, and with self-transcendence, unfolding into specific ways of elaboration of religiosity. Rooted, then, in this intrinsic move of spirituality, the propeller of meaning, the belief in miracles can have different destinations, even those described as positive or negative in the Pargament et al. (2000) scale. So, for example:

a) On acquiring a transforming character of oneself or one's own life, modes of belief in the miracle can be seen unfolding, corresponding to the description of the first positive factor contemplated in the referred scale;
b) On mobilizing actions that result in offering or obtaining concrete help from others (such as an individual, family, institution, or specific groups), will result in aspects described in the second, third, and sixth positive factors of that scale;
c) On strengthening one's self-help potential or personal growth resulting from a deep search for contact with oneself, based on an unshakable faith and in the certainty of the support of a greater force, also promotes a positive position toward God and the consequences contemplated in the fourth, fifth and seventh positive factors of the same scale;
d) On manifesting itself as a form of temporary distancing from the problem to gain time and remodel the desire for health and healing, it also favors a change in perspectives, as seen in the eighth positive factor of the scale in question;
e) However, on the other hand, when it crystallizes in certain attitudes that deny reality or in interminable supplications, it can result in a disappointment with religion itself, if personal desire, of a narcissistic nature, and projected on religion and/or perspective of the miracle of healing does not take place, characterizing one or more of the four factors of religious coping described as negative described in the scale originally developed by the aforementioned authors (Pargament et al., 2000).

The model developed by Shinall et al. (2018) can also be understood even better from this broader perspective, where how faith in the miracle is manifested - or the expectation of its occurrence, in situations of very bleak diagnoses – is related to this line of continuity between the notions of spirituality, religiosity, and religion. Thus, for example, the modes of manifestation in the miracle may have destinations characterized as “harmless” or lead to a kind of “shattering of hope” if they are sustained only in a rigid, crystallized religious perspective, confused with the most narcissistic desires themselves, and without spaces for the propulsion to re-signification and for the rediscovery of meanings in the disease itself, which would be driven by spirituality. Or, even as a movement anchored on religion, the belief in the miracle can be a driving force for greater or lesser “integration,” also depending on the way the professionals themselves conceive the religiosities of the patients themselves and react to them in the hospital context, many sometimes disagreeing with the forms of meaning sought through them. Depending, then, on this mediation between the impulse to search for meaning, as something that is at the base of belief, and respect for the different ways of achieving it (whether in one religion or another; whether in religion and/or in science), the relationships between patients and professionals may contribute, in a greater or lesser extension, to a more or less “strategic” destiny of the belief in the miracle in the health context.

In the same direction, one can understand the effectiveness of the model proposed by Bibler et al. (2020). After all, a dynamic understanding of the relationships between these three dimensions—religion, religiosity, and spirituality—can open new paths in the attitude of the professionals themselves when dealing with patients' religiosities, expanding their respective abilities to embrace the dimension of meaning that accompanies them, favoring a more “integrated” approach between the religious/spiritual perspective of the miracle and the perspective of medical care. Such skills will be essential to better relationships both with those who present themselves as true “proxies” and dependent on their religious community, as well as with the “adaptable,” whose faith characteristics are not necessarily linked to specific religions but are also moved by a spirituality that sustains their respective beliefs in the miracle.

After all, from this broader perspective, belief in miracles is no longer seen as violating natural laws or technical knowledge in medicine (Doessy, 2018), but recognized as a genuine manifestation of the desire of living with meaning (Saad and Medeiros, 2018). This is in keeping with a vision of health also more far-reaching and not restricted only to organic or psychic wellbeing as Dejours (1986, p. 9, *emphasis ours*) points out:

“Health is certainly not psychic wellbeing. **Health is when having hope is permitted**. It can be seen that this changes things a bit. What makes people live is, above all, their desire; this is an achievement of psychiatry and psychosomatics. The real danger exists when there is no more desire when it is no longer possible.”

Seen from this angle, we recognize the belief in miracles, a propelling act of hope in a less painful future. Under this perspective, the experience of “praying for a miracle” initially occurs as an act of openness to life instead of immediate closure in a gloomy perspective existentially imposed by a technical and scientistic perspective. This openness is healthy, from the psychological and existential point of view, as far as, through it, the necessary time is gained so that the painful realities of a gloomy diagnosis like fetal congenital malformation (FCM) in pregnant women or child cancer may be re-signified in the psyche of the expectant mother, of the child, or its family members. This understanding, however, is only possible considering a flexible and integrating model of conceptualization, where both the distinctions and connections between spirituality, religiosity, and religion are taken into consideration.

In a model where spirituality refers to the impulsion of meaning and the search of answers to human existence and its vicissitudes, the intentionality and human capacity of reflection about him or herself, and about the experience in the surrounding world is genuinely qualified. As such, it can refer to or be based on religiosity whose answers of meaning are rooted in the transcendent, and they may or may not be linked to a particular system of sharing under the form of doctrine, dogma, and/or institutionalization. Belief in the possibility of a miracle in the face of bleak diagnoses can then be a mechanism of positive or negative religious coping according to the degree of subjective or intersubjective flexibility that circulates between these three dimensions. So, belief in a miracle, represented by the act of praying for one, appears as a positive component of SRC, when nurtured by a spirituality that results in the search for meaning, sparking off hope to cope with desolating sentiments such as grief, guilt, or anger about pregnancy with FCM or having a child with cancer. The hope of a miracle, for expectant mothers with FCM or family members of carcinogenic children can be a way of avoiding the reality without denying it, constituting part of a process of psychic adaptation to suffering and culminating in the search for the meaning of the lived experience, often represented in the apogee of the re-signification, through maternal love. It can also be a kind of network support where family members nurture the hope of the patient who is still under the harsh impact of the gloomy diagnosis. Thus, through their prayers for a miracle, they strengthen affective bonds and reciprocal support among themselves until they are more emotionally prepared to cope with the limits or complete impossibility of reversing the diagnosis. This can be a much more positive process when it is prone to be elaborated and preparing for a re-signification of the meaning of the diagnosis for the life of all the people involved.

Nevertheless, the negative aspect of belief in miracles is also observed in clinical practice when the expectant mother or family member of the child with cancer (or some gloomy diagnosis) insistently seeks what is an improbable, cure, even in the face of medical evidence for such. When such a search is based only on dogmas that some religious institutions adopt and diffuse, being static and grounded on a linear interpretation of “miracle,” serving traditions or orthodoxies inclined to religious fundamentalism, it can be problematic and impervious to the process of re-signification over time. In this situation, the religiosity of the expectant mother or family member is not properly focused on transcendence, but on the pragmatic result desired by him or her, in many cases, rooted in a religious doctrine. In other words, the belief in the possibility of a miracle in the face of bleak diagnoses can be a mechanism of positive or negative religious-spiritual coping according to the degree of the subjective flexibility or inflexibility that permeates the dimensions of spirituality, religiosity, and religion. Or still, when rooted in a perspective where the dimension of meaning is not kept open, capable of incorporating the suffering as replete with signification through the exercise of auto reflexivity. In these cases, belief in miracles would be anchored on a more reductionist vision of the transcendent taken as a dimension at the mercy of the individual and emotional necessities. Its negative impact granted its egocentric and (de)negating character of the surrounding reality, flows consequently. It is exactly in these cases that arise the necessity of developing skills and socio-cultural competences in the psychology of religion or applied spirituality by health professionals and inter or multidisciplinary teams to handle the question. This would be the subject of a future paper.

## Author Contributions

MF and ML intellectually conceived the paper. MF wrote the paper with the help of ML and EN. ML and EN revised the paper. EN did the translation and corrections. All authors contributed to the article and approved the submitted version.

## Funding

This article was funded by Research Support Foundation of the Federal District (the *Fundação de Apoio à Pesquisa do Distrito Federal* [FAP-DF]), Brasília/Brazil, by funds made available through Public Call 11/2022 - Public Selection of Proposals for Financial Support for Publication in Scientific Journals.

## Conflict of Interest

The authors declare that the research was conducted in the absence of any commercial or financial relationships that could be construed as a potential conflict of interest.

## Publisher's Note

All claims expressed in this article are solely those of the authors and do not necessarily represent those of their affiliated organizations, or those of the publisher, the editors and the reviewers. Any product that may be evaluated in this article, or claim that may be made by its manufacturer, is not guaranteed or endorsed by the publisher.
